# Geriatric nutritional risk index improves risk scoring for mortality after TAVR compared to established scores

**DOI:** 10.3389/fcvm.2026.1774104

**Published:** 2026-03-19

**Authors:** Johannes Boehm, Andrea Amabile, Hendrik Ruge, Martin Bichler, Stefan Holdenrieder, Melchior Burri, Markus Krane

**Affiliations:** 1 Department of Cardiovascular Surgery, Institute Insure, TUM University Hospital - German Heart Center, TUM School of Medicine & Health, Technical University of Munich, Munich, Germany; 2 Division of Cardiac Surgery, Department of Cardiothoracic Surgery, University of Pittsburgh, Pittsburgh, PA, United States; 3 UPMC Heart and Vascular Institute, University of Pittsburgh Medical Center, Pittsburgh, PA, United States; 4 Institute for Laboratory Medicine, German Heart Center, Technical University of Munich, Munich, Germany; 5 DZHK (German Center for Cardiovascular Research), Partner Site Munich Heart Alliance, Munich, Germany; 6 Division of Cardiac Surgery, Department of Surgery, Yale School of Medicine, New Haven, CT, United States

**Keywords:** 30-day mortality, geriatrics, GNRI, nutritional scores, outcome analysis, risk scoring, TAVR

## Abstract

**Background:**

Risk scoring prior TAVR is based on the EuroSCORE II (European System for Cardiac Operative Risk Evaluation) and the STS-score (Society of Thoracic Surgeons) which are complex, and partly prone to investigator bias. The geriatric nutritional risk index (GNRI) can be calculated by five parameters via publicly available formula (age, height, weight, sex, serum albumin), whereas EuroSCORE II and the STS-score require 21 and 69 variables, respectively. The study compares the efficiency of GNRI in predicting 30-day mortality compared to the EuroSCORE II and the STS-score. Furthermore, GNRI risk classes were analysed in the long-term.

**Methods:**

3.470 consecutive patients who underwent TAVR between 2010 and 2023 at our institution were analysed. GNRI calculation produces a linear parameter that can be divided in four risk groups.

**Results:**

ROC (receiver operating characteristic) curve analysis demonstrated no difference in predicting 30-day mortality between GNRI vs. EuroSCORE II (AUC = 0.72 vs. 0.69, *p* = 0.3) and GNRI vs. STS-score (AUC = 0.72 vs. 0.72, *p* = 1.0). The Hosmer-Lemeshow test indicated good calibration for the GNRI model (*p* = 0.3793). After adjustment for preoperative demographic characteristics, Cox regression analysis for overall survival after TAVR reveals for the major risk group [21 patients; HR = 4.624; CI95%(2.881–7.422); *p* < 0.0001], the moderate risk group [198 patients (5.7%), HR = 2.201; CI95%[1.821–2.660]; *p* < 0.0001], and the low risk group [452 patients (13.0%); HR = 1.831; CI95%[1.597–2.1]; *p* < 0.0001], respectively.

**Conclusions:**

The GNRI is an objective publicly available score that simplifies risk assessment prior TAVR without any loss of precision compared to the EuroSCORE II and the STS-score.

## Introduction

Preoperative risk assessment remains a core clinical challenge in cardiovascular medicine and is subject of extensive research (1–10). Risk assessment is primarily based on two well established scores for cardiac surgery, that have become gold standards over the years to predict mortality after surgery: the European System for Cardiac Operative Risk Evaluation (EuroSCORE) II and the Society of Thoracic Surgeons (STS) predicted risk of mortality (1, 3, 11).

The EuroSCORE, primarily derived from European databases, has been developed by Nashef et al. (4) and has been steadily modified over the years to evolve into EuroSCORE II (5). The STS Score has been developed from the Society of Thoracic Surgeons database to predict outcome after cardiac surgery (11), but has become one of the cornerstones of risk assessment in TAVR as well (12, 13). Both scores yield excellent results but are notably complex. Data entry must be performed manually on a web-based interface, and, to our knowledge, the precise algorithms behind these scores are not publicly accessible. Simplification would bring substantial benefits for research and clinical practice.

In contrast, the novel Geriatric Nutritional Risk Index (GNRI) was developed in an entirely different context (14). It aims to predict mortality in elderly patients (aged >65 years) using only five objectively measurable clinical parameters: age, height, weight, sex, and the serum levels of albumin. The underlying formula, which produces a continuous GNRI value, is straightforward, easy to apply and publicly accessible. GNRI has demonstrated excellent prognostic power in various clinical settings, predicting mortality in cancer patients, as well as those undergoing coronary interventions or TAVR.

However, GNRI has not yet been tested in TAVR patients against the best currently available scores to predict mortality after TAVR, EuroSCORE II and the STS score. The current study evaluates the potential of GNRI in predicting early mortality following TAVR compared to the EuroSCORE II and the STS score in a large single-centre experience.

## Methods

The current study evaluates the potential of GNRI in predicting early mortality following TAVR compared to the EuroSCORE II and the STS score in a large single-centre experience.

### Patients

**Figure 1:**
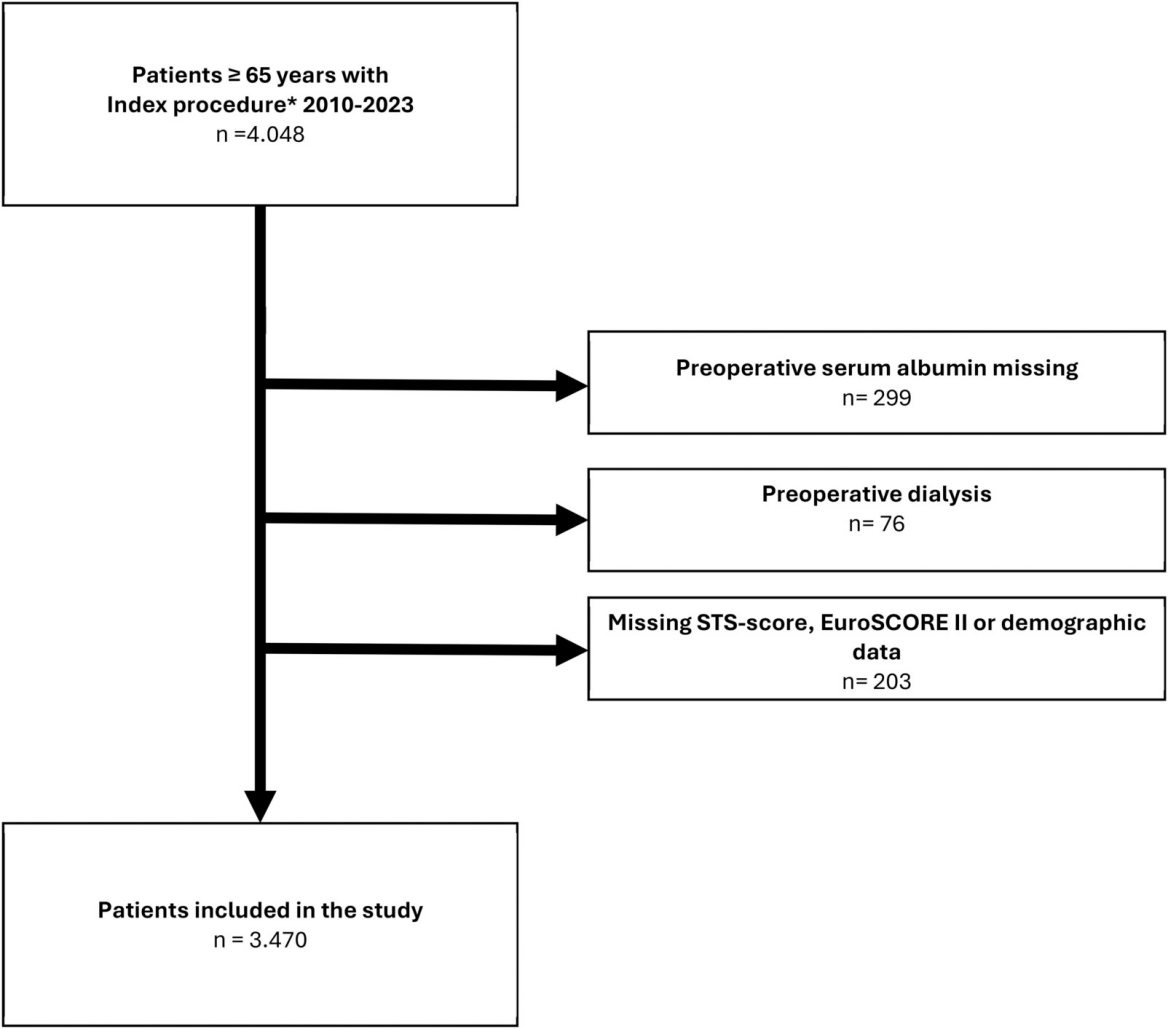
Patient flow chart.

We included all consecutive patients who underwent a TAVR procedure at the German Heart Center in Munich between 2010 and 2023. Data were obtained from an ongoing quality assessment program. All medical records, including operative reports, in-hospital and outpatient clinical notes were reviewed. After the exclusion of patients with missing data and patients on dialysis, a total of 3.470 patients were included in the study (Figure 1). Serum levels of albumin were measured prior to surgery, according to our institutional standard.

### Approvement

The study protocol was approved by the Ethics Committee of the Medical School of the Technical University of Munich (2025-280-S-CB). Individual patient consent was waived, because it was not practicable to obtain consent from large numbers of patients for a retrospective chart review study.

### GNRI

The score was calculated following the paper by Bouillanne et al. that introduced the GNRI (14). The following formula was applied: GNRI = [1.489 × serum albumin (g/L)] + [41.7 × (current body weight/ideal body weight)]. GNRI classes were defined according to former research: Major risk (GNRI < 82), moderate risk (GNRI =  ≥ 82 and <92), low risk (GNRI = 92 to ≤98) and minimal risk (GNRI > 98).

### Survival

All-cause mortality at day 30 and during the follow-up was calculated, and Kaplan–Meier analysis was applied as appropriate. Cox regression analysis was performed for demographic parameter and the first three risk classes of GNRI (major risk, moderate risk, and low risk).

### Statistics

Statistical analysis was performed by using IBM SPSS Statistics 28.0 software (IBM Corp, Armonk, NY USA) and R Version 4.2 (R Core Team. 2024, R Foundation for Statistical Computing). Data were presented as mean ± standard deviation for continuous variables and number (%) for categorical variables. Analyses of variances (ANOVA) testing was used to compare mean values, and Chi-square- and Kruskal—Wallis tests were used as appropriate. Survival rates were calculated using Kaplan–Meier methods. Statistical significance was set at 0.05. Receiver Operating Characteristic (ROC) curves were used to assess the predictive power for 30-day mortality of the GNRI, the STS score, and the EuroSCORE II. The AUC (area under the curve) was analyzed for all scores. AUĆs were compared using the Delong test. Calibration plots for GNRI, EuroSCORE II, and STS score were drawn using the Hosmer-Lemeshow test.

## Results

**Figure 2:**
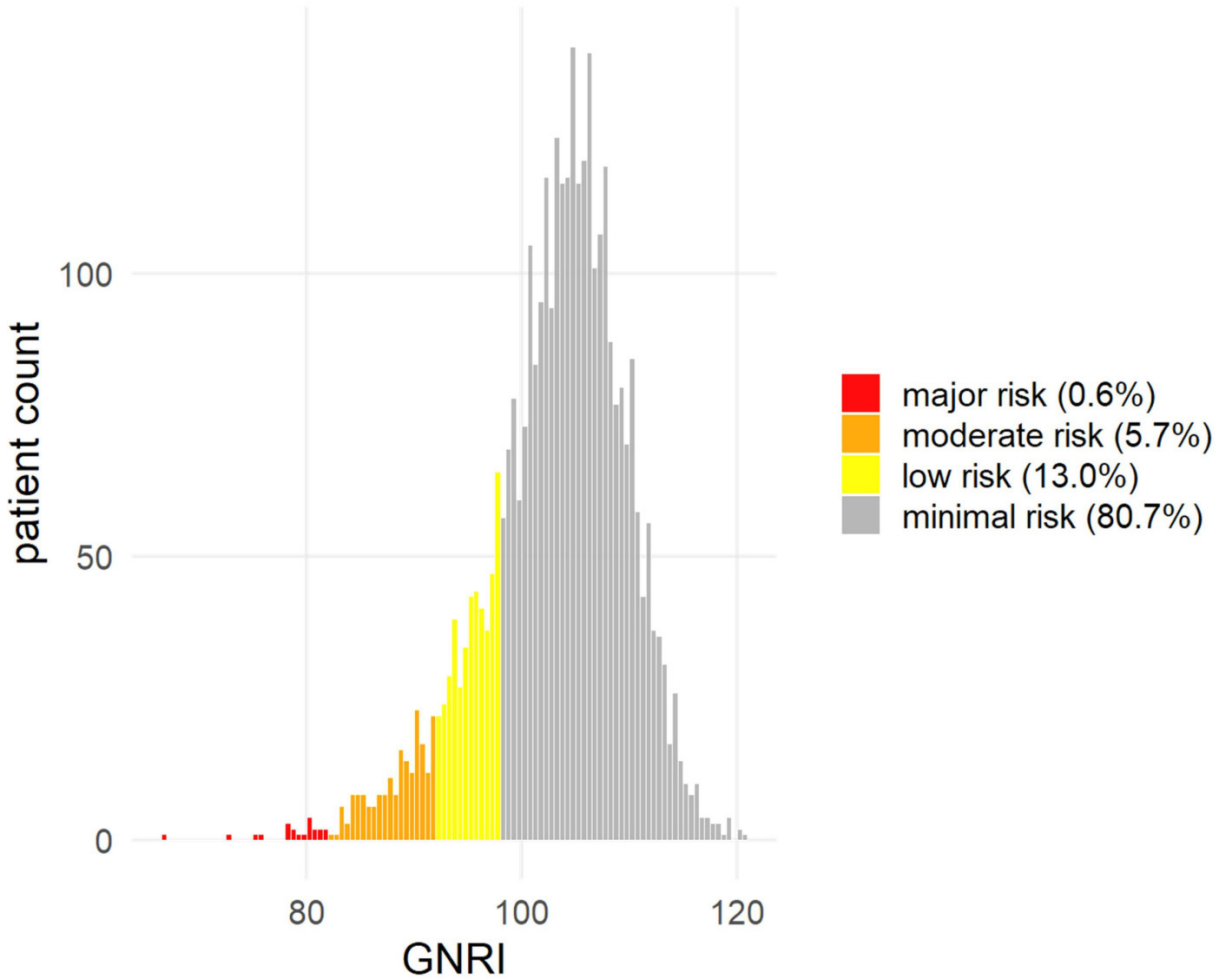
Frequencies of GNRI classes.

A total of 3.470 patients were included. Patients were 80.4 ± 5.9 years old. The mean EuroSCORE II was 5.2 ± 7.6, and STS score was 4.2 ± 3.7. GNRI formula creates a linear parameter, that can further be divided in four GNRI categories: 21 patients were at major risk, 198 (5.7%) patients were at moderate risk, 452 (13%) patients were at low risk, and 2.799 patients (80.7%) patients were at minimal risk (Figure 2). A probability plot of GNRI values is given in Figure 2. Demographics are given in Table 1 and the parameters differed between the groups as expected (Table 1).

**Table 1:** Demographics.

Label	Major risk	Moderate risk	Low risk	Minimal risk	*P* value
n	21	198	452	2,799	
Demographics					
Age	83 [81–85]	82 [78–86]	82 [78–85]	81 [77–84]	<0.001
Male sex	11 (52.4%)	106 (53.5%)	218 (48.2%)	1,483 (53.0%)	0.3
BMI	21 [19–25]	25 [21–28]	25 [22–29]	27 [24–30]	<0.001
Arterial hypertension	16 (76.2%)	169 (85.4%)	403 (89.2%)	2,557 (91.4%)	0.002
Diabetes	5 (23.8%)	54 (27.3%)	143 (31.6%)	827 (29.5%)	0.6
Family history	1 (4.8%)	21 (10.6%)	60 (13.3%)	575 (20.5%)	<0.001
Hyperlipidemia	6 (28.6%)	112 (56.6%)	280 (61.9%)	2,012 (71.9%)	<0.001
Smoking	7 (33.3%)	55 (27.8%)	130 (28.8%)	811 (29.0%)	1.0
Periperal artery disease	2 (9.5%)	29 (14.6%)	81 (17.9%)	340 (12.1%)	0.007
COPD^a^	7 (33.3%)	52 (26.3%)	97 (21.5%)	472 (16.9%)	<0.001
Creatinine (mg/dL)	1.3 [1.0–1.6]	1.2 [0.9–1.6]	1.1 [0.9–1.5]	1.1 [0.9–1.3]	<0.001
Procedural Details					
Access					0.3
direct aortic via ministernotomy	0 (0.0%)	3 (1.5%)	8 (1.8%)	42 (1.5%)	
subclavia	2 (9.5%)	4 (2.0%)	17 (3.8%)	62 (2.2%)	
transapical	3 (14.3%)	18 (9.1%)	52 (11.5%)	290 (10.4%)	
transfemoral	16 (76.2%)	173 (87.4%)	375 (83.0%)	2,405 (85.9%)	
Emergency surgery	2 (9.5%)	10 (5.1%)	3 (0.7%)	8 (0.3%)	<0.001
Prior cardiac surgery	5 (23.8%)	25 (12.6%)	62 (13.7%)	408 (14.6%)	0.5
Scores and Mortality					
EuroSCORE II	9.7 [8.0–18.2]	10.9 [6.3–19.4]	7.6 [4.2–14.3]	4.6 [2.5–9.2]	<0.001
STS	6.1 [3.5–9.0]	4.7 [2.9–7.4]	4.1 [2.5–6.1]	3.0 [2.0–4.6]	<0.001
Base Hospital Mortality	6 (28.6%)	7 (3.5%)	12 (2.7%)	49 (1.8%)	<0.001
30-Days Mortality	7 (33.3%)	27 (13.6%)	25 (5.5%)	65 (2.3%)	<0.001

^a^ Chronic obstructive pulmonary disease.

### Survival analysis

Mean follow-up time was 2.126,95 ± 37.63 days [CI 95% (2.053,2–2.200,7)], median follow-up time was 1.930 ± 48.7 days [CI 95% (1.834,6–2.025,4)]. 30-day mortality in these four groups were (33.3%), (13.6%), (5.5%), and (2.3%) in the minimal risk group, respectively (*p* < 0.0001) (Table 1). After adjustment for preoperative demographic characteristics, Cox regression analysis for overall survival after TAVR revealed the following for the different GNRI classes: Major risk group [21 patients; HR = 4.624; CI95%(2.881–7.422); *p* < 0.0001], the moderate risk group [198 patients (5.7%), HR = 2.201; CI95%[1.821–2.660]; *p* < 0.0001], and the low risk group [452 patients (13.0%); HR = 1.831; CI95%[1.597–2.1]; *p* < 0.0001]. It should be highlighted that the 3 tested GNRI classes have higher odds for mortality than any other preoperative parameter like age [HR = 1.063; CI 95% (1.053–1.073); *p* < 0.0001], chronic obstructive pulmonary disease [HR = 1.273; CI 95% (1.119–1.449); *p* < 0.0001] or creatinine [HR = 1.453; CI 95% (1.344–1.571); *p* < 0.0001] (Table 2).

**Table 2:** Cox regression analysis on mortality for preoperative demographic parameters.

Variable	B^a^	SE^b^	HR^c^	Lower	Upper	*p*
GNRI^d^- Major risk	1.531	0.241	4.624	2.881	7.422	<0.001
GNRI—Moderate risk	0.789	0.097	2.201	1.821	2.660	<0.001
GNRI—Low risk	0.605	0.070	1.831	1.597	2.100	<0.001
Age	0.061	0.005	1.063	1.053	1.073	<0.001
Female gender	−0.100	0.055	0.905	0.813	1.007	0.066
Body mass index	−0.002	0.006	0.998	0.987	1.010	0.768
Diabetes	0.315	0.058	1.370	1.223	1.534	<0.001
Hyperlipidemia	−0.003	0.057	0.997	0.892	1.114	0.955
Arterial hypertension	−0.063	0.090	0.939	0.787	1.119	0.481
Smoking	0.050	0.059	1.051	0.937	1.180	0.393
PAVD^e^	0.313	0.072	1.367	1.187	1.575	<0.001
COPD^f^	0.242	0.066	1.273	1.119	1.449	<0.001
Creatinine	0.373	0.040	1.453	1.344	1.571	<0.001

^a^ Regression coefficient.

^b^ Standard error.

^c^ Hazard ratio.

^d^ Geriatric nutritional index.

^e^ Peripheral artery disease.

^f^ Chronic obstructive pulmonary disease.

**Figure 3:**
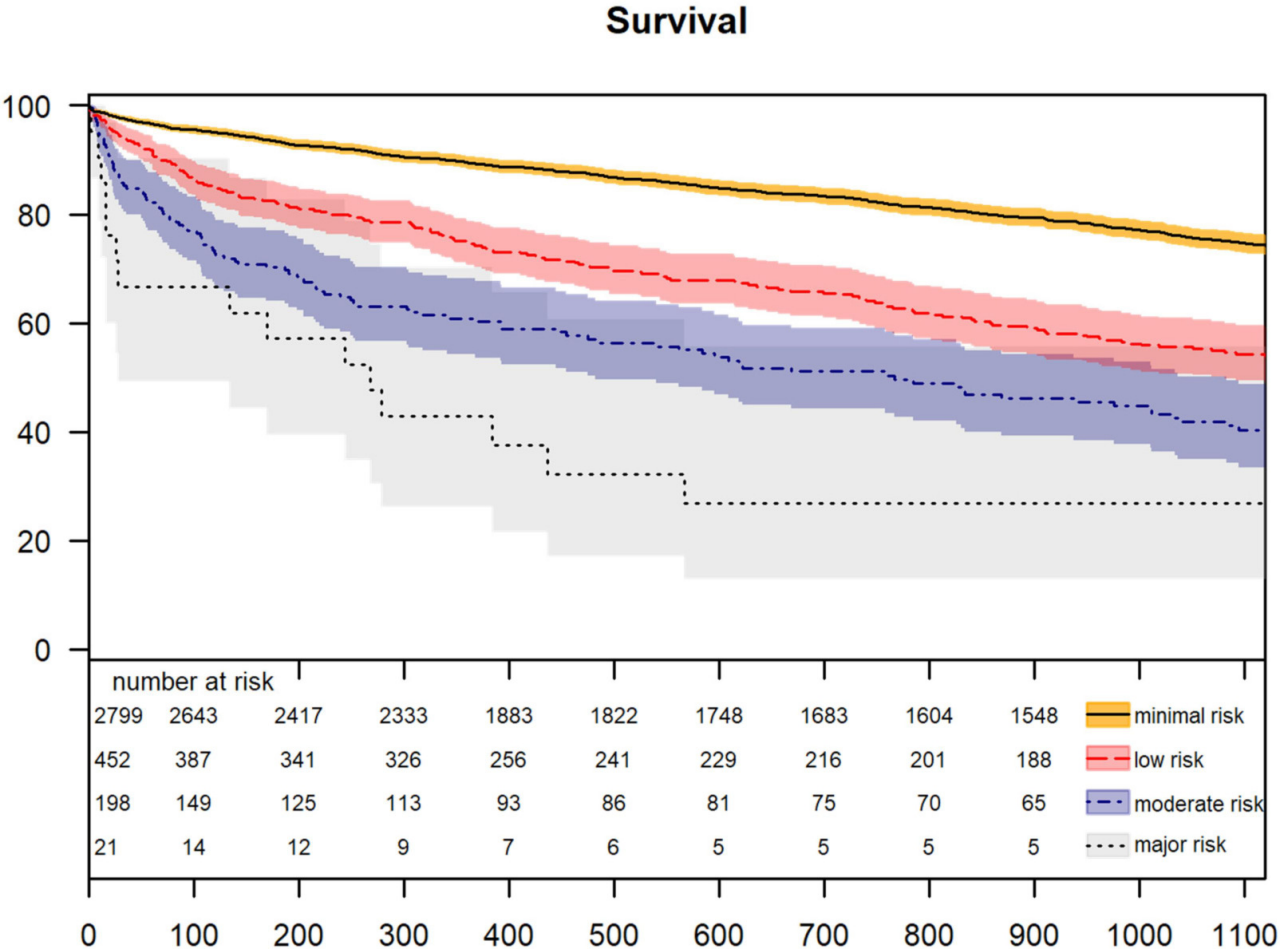
Survival.

GNRI classes showed significantly different survival rates as demonstrated by Kaplan–Meier analysis. Survival estimates for 1- and 5-years survival were estimated for the major risk group 42.9% ± 10.8 and 13.4% ± 8.3, for the moderate risk group 60.8% ± 3.5 and 27.5% ± 3.8, for the low risk group 74.5% ± 2.1 and 34.1% ± 2.8, and for the minimal risk group 92.7% ± 0.05 and 57.9% ± 1.2, respectively (Figure 3).

### Predictive power for 30-day mortality

**Figure 4:**
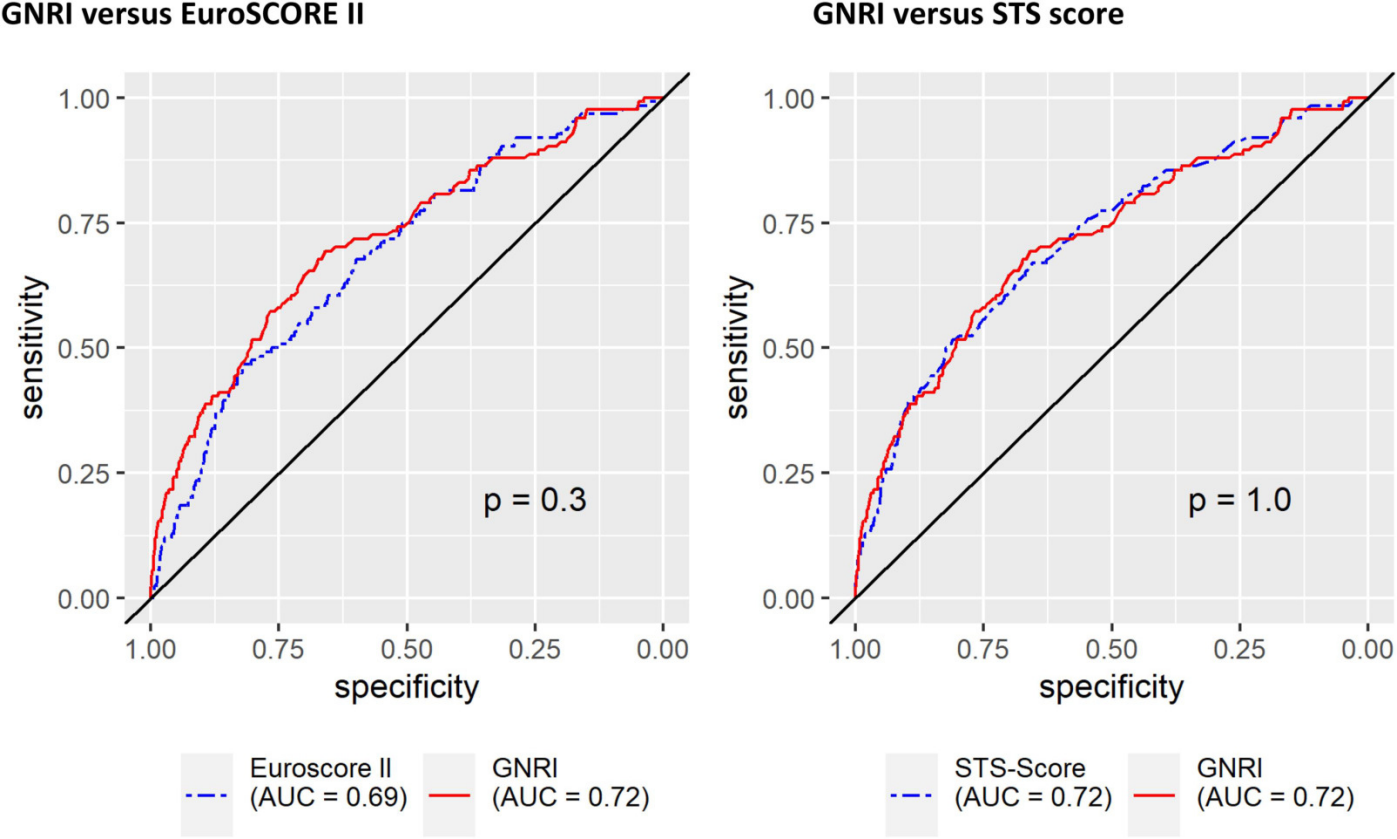
Prediction of all-cause 30-day mortality: GNRI vs EuroSCORE II and GNRI vs STS score.

**Figure 5:**
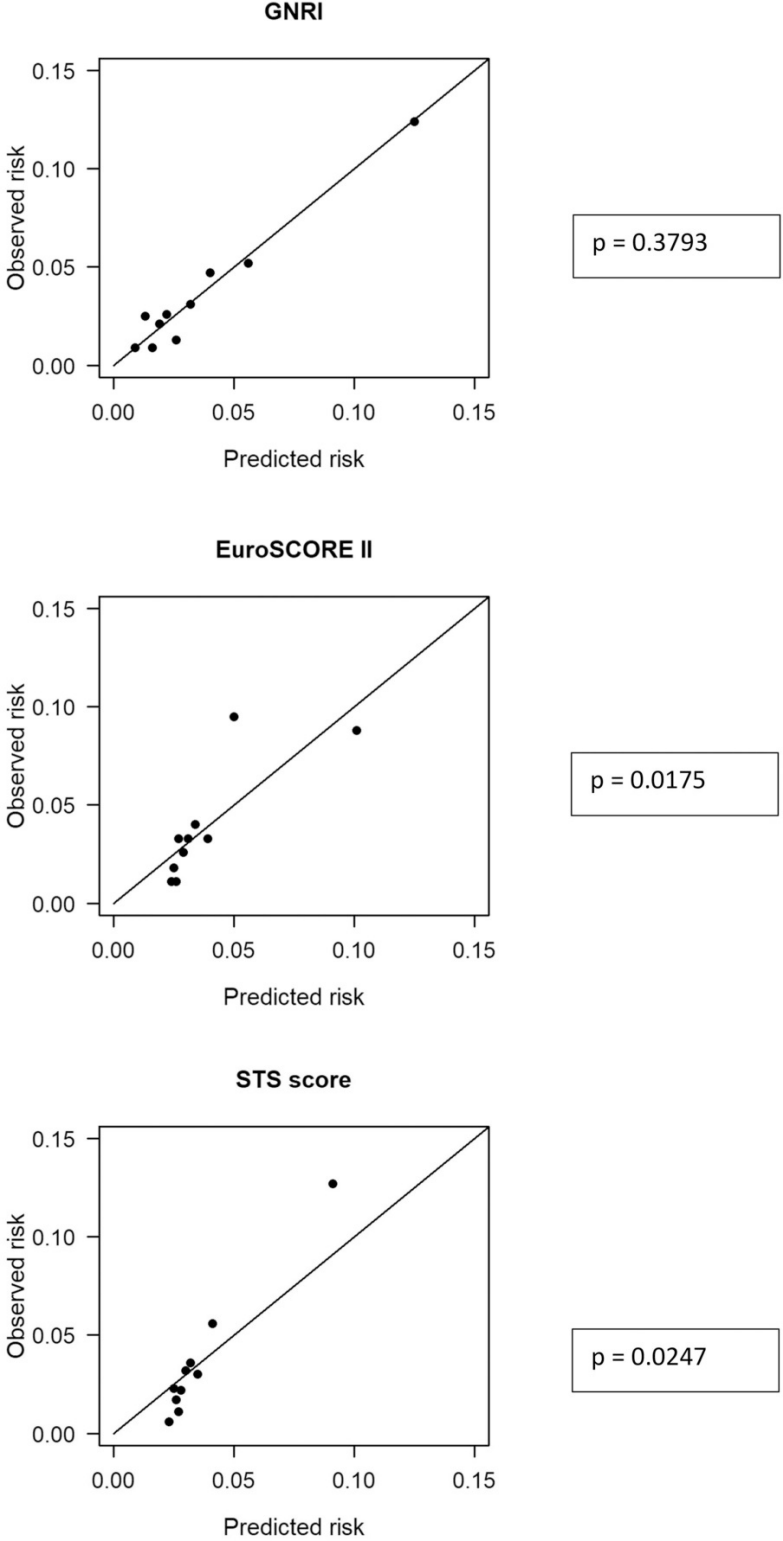
Calibration plots for GNRI, EuroSCORE II, and STS score.

Receiver operating risk analyses were performed to compare the predictive power of 30-day mortality of GNRI vs. the EuroSCORE II and of GNRI vs. the STS predicted risk of mortality. The GNRI turned out to be as precise as the EuroSCORE II with an area under the curve (AUC) of 0.72 for the GNRI vs. 0.69 for the EuroSCORE II and 0.72 vs. 0.72 for the STS predicted risk of mortality (*p* = 1.0; Figure 4). Calibration plot for GNRI according to Hosmer-Lemeshow sees the GNRI as the best-calibrated model (*p* = 0.3793); calibration plots for the EuroSCORE II revealed *p* = 0.0175, and for the STS score *p* = 0.0247 (Figure 5).

### Data requirements and complexity of GNRI vs. STS score and EuroSCORE II

**Figure 6:**
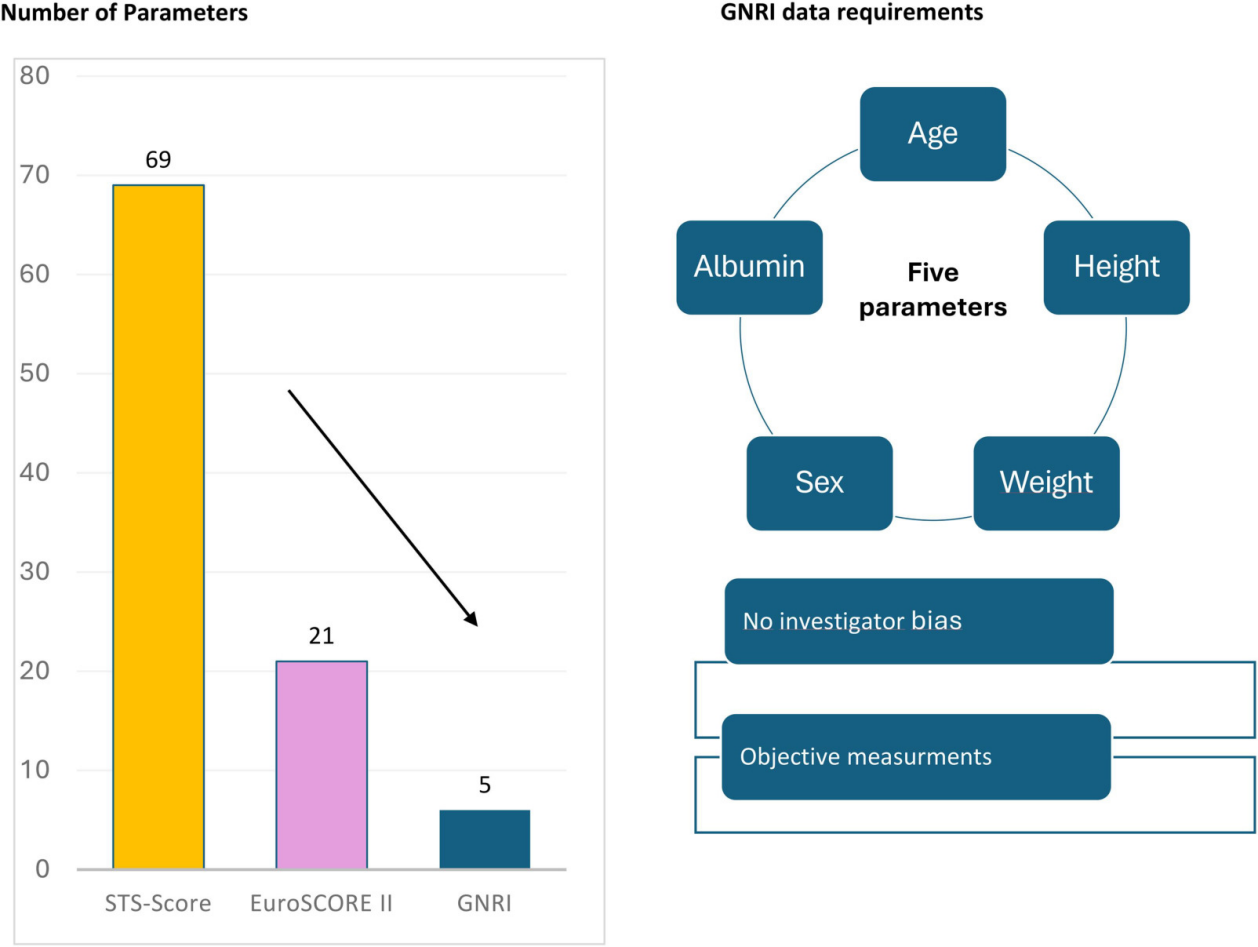
Data requirements for individual score calculation.

Data requirements and complexity differ substantially between the scores. Figure 6 gives the number of required parameters per score and indicates the number of parameters with potential biases. For the STS score, the parameters race, payment, liver disease, heart failure, and NYHA classification were counted as not objectively measured or prone to potential biases. For the EuroSCORE II, parameters like poor mobility, CCS angina class, NYHA classification, and urgency of operation were counted as prone to biases. Previous cardiac surgery and recent myocardial infarction cover a huge clinical spectrum and are therefore not objectively measured.

## Discussion

Risk stratification remains a core clinical challenge, and the requirements for an ideal scoring system are exceptionally high. Extensive research is currently being conducted to optimize the timing of surgery (15, 16) or expand the indications to include lower-risk groups (17). These efforts would greatly benefit from optimal risk scoring. An optimal score must be easy to perform, cost-effective, widely accessible, and—most importantly—highly accurate. In the specific context of TAVR procedures, no such risk model has been identified so far (10, 13, 18).

This study demonstrates that the established scores—EuroSCORE II and STS predicted risk of mortality—exhibit comparable predictive power for 30-day mortality in a large, real-world cohort spanning a 14-year observer period. Remarkably, the much simpler GNRI achieves equivalent predictive performance for short-term mortality (Figure 4). Moreover, GNRI-based risk categories were independently associated with survival in multivariate Cox regression analysis (Table 2), outperforming standard demographic and perioperative parameters. This prognostic value extended to long-term outcomes: patients categorized as moderated or major risk according to GNRI exhibited significantly impaired long-term survival (Figure 3). Given that the GNRI requires only a handful of objective, routinely collected variables, its use could dramatically simplify preoperative risk stratification in TAVR—free from any investigator bias and easily applicable in retrospective analyses.

The Geriatric Nutritional Risk Index (GNRI), originally developed to estimate mortality risk in individuals aged 65 years and older, has been validated across a range of clinical settings. The rationale for these studies is rooted in the well-documented negative impact of malnutrition on cardiovascular outcomes, its association with frailty, and the adverse prognostic implications of compromised nutritional status (19–21). Lower GNRI levels are linked to frailty and increased cardiovascular mortality (22). In cardiovascular medicine, research has primarily focused on its predictive value in heart failure (23–27). The GNRI has also been validated as a predictor of mortality in patients with acute decompensated heart failure (28), even when assessed at hospital discharge (29), and for predicting 30-day mortality after cardiac surgery using cardiopulmonary bypass (30).

Current research found that the nutritional status predicts poor survival after TAVR. Mas-Peiro et al. found the GNRI to be a stronger prognostic indicator than other nutritional factors in a smaller TAVR cohort (31), and similar findings that a nutritional score predict the outcome after TAVR was shown by Okuno et al. (32). Focussing on mid-term outcome, i.e., >1 years, poor GNRI values are associated with poor outcome after TAVR (33–35), but GNRI was not compared to established risk scores in predicting short-term mortality after TAVR. In patients undergoing PCI for acute coronary syndrome, several nutritional scores, including GNRI, have been shown to be predictive for major adverse cardiac events (MACE) within one year after the procedure (36), and a subsequent study, combining GNRI with another index, the Systemic Inflammatory Index, resulted in superb predictive power for MACE within one year after intervention (37).

### Score complexity and GNRI as alternative

The complexity of EuroSCORE II and the STS score extends beyond the sheer number of required inputs— 21 and 69 variables, respectively (Figure 6). Many of these parameters are either not objectively measurable (e.g., angina or NYHA class) or imprecisely defined across a broad clinical spectrum (e.g., “recent myocardial infarction”). Additionally, some parameters are not precise when covering a huge clinical spectrum like recent “myocardial infarction” or “previous cardiac surgery” in the EuroSCORE II (5) or “race” in the STS score (3). Moreover, the exact formulas for both scores are not publicly available.

Despite their complexity, EuroSCORE II and the STS score share several core variables, i.e., symptoms related parameters like NYHA classifications or laboratory values like creatinine. However, neither incorporates preoperative albumin levels—a key component of the GNRI. Incorporating albumin, a highly standardized and unbiased biomarker, may be critical to the predictive strength of the GNRI. Free from any potential bias, albumin measurement is highly standardized, and albumin levels have been shown to have a predictive impact of their own prior surgery in a variety of clinical settings, i.e., surgery for infective endocarditis (38), or coronary interventions (39). However, it is important to note that albumin priming of cardiopulmonary bypass has not been shown to improve outcomes (40). This reinforces the importance of baseline, preoperative albumin measurements as a key prognostic factor.

Nonetheless, the AUC of the GNRI was not superior to any of the established scores. GNRI main advantage over the current gold standard lies instead in its simplicity, requiring only a small amount of data and relying entirely on objective measurements. Furthermore, GNRI should be interpreted with caution in severe infections, such as endocarditis, as albumin levels in these situations primarily reflect inflammatory activity rather than nutritional status, and that GNRI is probably not the rate choice in those cases.

As this analysis focuses exclusively on all-cause 30-day mortality, operative mortality, perioperative complications, and cause-specific mortality (including MACCE-related deaths) were not further differentiated.

In conclusion, the GNRI shows significant promise as a simplified, objective tool for risk stratification in patients undergoing TAVR. Future studies are warranted to validate the findings of this study and to explore the integration of GNRI into routine clinical risk assessment algorithms.

### Perspectives

GNRI predicts 30-day mortality as accurate as the STS-score and the EuroSCORE II, and thereby offers a substantial simplification of risk assessment prior TAVR without any loss of precision.

## Data Availability

The data underlying this article will be shared on reasonable request to the corresponding author. Requests to access these datasets should be directed to boehm.jo@me.com.
